# Retinal Microvascular Resistance Estimated From Waveform Analysis Is Significantly Higher in Diabetic Retinopathy

**DOI:** 10.1167/tvst.14.11.30

**Published:** 2025-11-19

**Authors:** Yuta Koyama, Yuki Nakano, Yukiko Miyoshi, Rie Osaka, Ayaka Hara, Kiyoshi Suzuma

**Affiliations:** 1 Department of Ophthalmology, Faculty of Medicine, Kagawa University, Miki-cho, Kagawa, Japan

**Keywords:** diabetic retinopathy, retinal blood flow, LSFG, MBR, TCR

## Abstract

**Purpose:**

Diabetic retinopathy (DR) is a typical complication in patients with diabetes. This study aimed to compare retinal blood flow and vascular resistance between eyes with DR and healthy eyes using laser speckle flowgraphy (LSFG).

**Methods:**

In total, 50 normal eyes and 87 DR eyes were examined at Kagawa University Hospital. LSFG was used to measure the mean blur rate (MBR) and total capillary resistance (TCR) of large vessels in the optic papilla. These values were compared across normal eyes and all eyes with DR, moderate nonproliferative diabetic retinopathy (NPDR), severe NPDR, and proliferative diabetic retinopathy (PDR). A TCR receiver operating characteristic (ROC) curve was plotted, and the diagnostic ability of the TCR for DR was determined using the area under the curve. The TCR cutoff value was determined using the Youden index.

**Results:**

No significant difference in MRB was observed between normal eyes and the other groups. TCR was significantly higher in all groups except the PDR group, compared to normal eyes. The TCR area under the ROC curve was 0.751, indicating moderate diagnostic accuracy for DR. Using the Youden index, the TCR cutoff value was 0.79 (sensitivity, 0.740; specificity, 0.701).

**Conclusions:**

Measuring TCR, in addition to MBR, as diagnostic markers provides more detailed pathological information regarding DR.

**Translational Relevance:**

Comparison of values between groups would be useful in predicting DR onset and stage progression.

## Introduction

Diabetic retinopathy (DR) occurs in patients with diabetes due to microvascular damage. The prevalence rates of retinopathy, proliferative diabetic retinopathy (PDR), and diabetic macular edema (DME) among Asian patients with diabetes have been reported as 19.9%, 1.5%, and 5.0%, respectively.^1^ DR is a severe clinical complication, with DME and vitreous hemorrhage leading to vision loss, visual field impairment, and, in advanced cases, the risk of blindness. Therefore, understanding the pathogenesis and predicting the progression of diabetic retinopathy are crucial.

Traditionally, color Doppler and laser Doppler techniques have been used to detect ocular blood flow.^2^^,^^3^ In the field of DR, laser Doppler techniques have been adopted to measure ocular blood flow.^4^^–^^6^ Retinal blood flow was reported to be reduced in eyes with diabetes without DR and mild nonproliferative DR (NPDR).^7^

**Figure 1.**
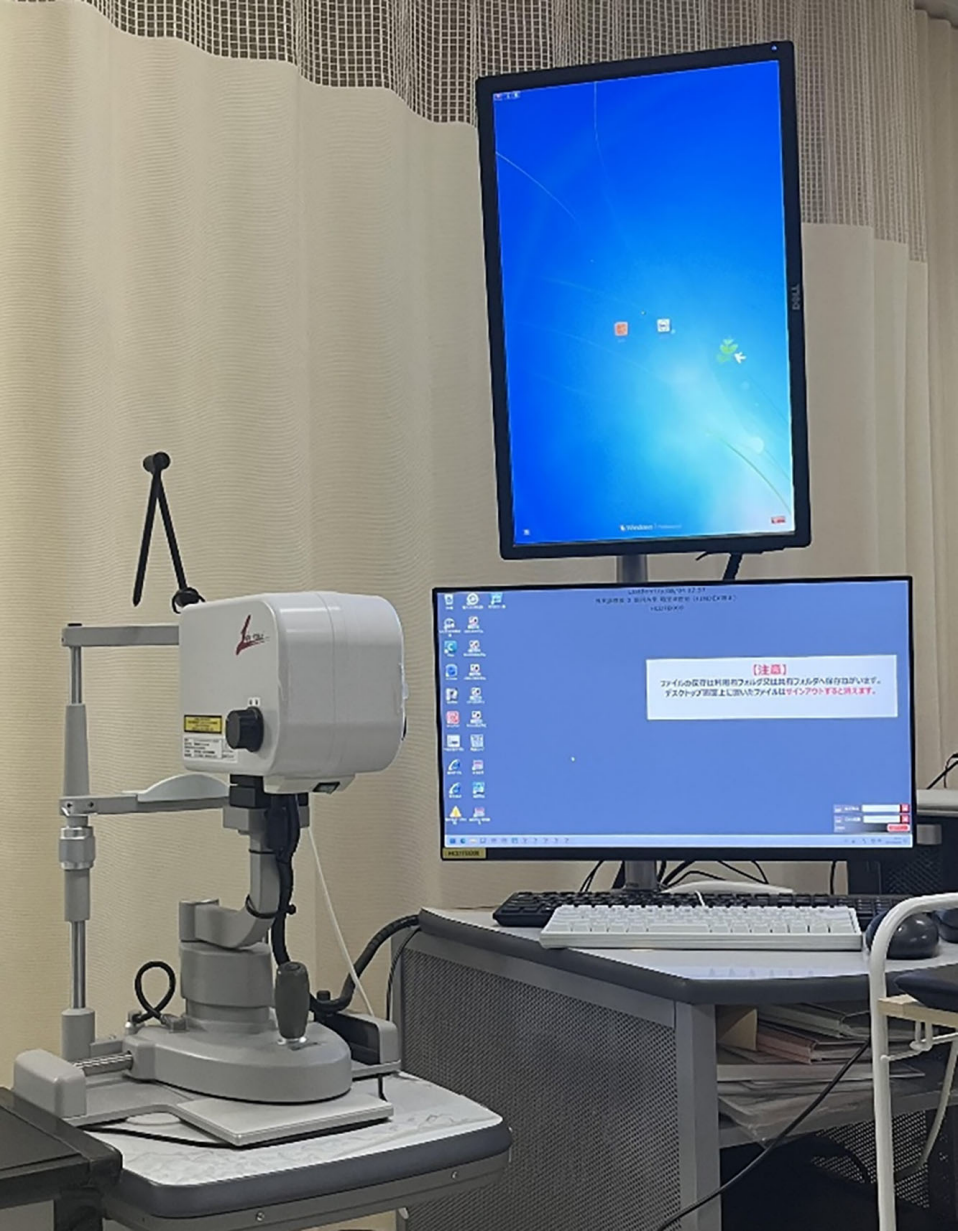
The appearance of the laser speckle flowgraphy device.

Recently, laser speckle flowgraphy (LSFG; Softcare, Fukuoka, Japan) has made it possible to image, measure, and analyze fundus blood flow distribution.^8^^–^^10^ Figure 1 shows the appearance of LSFG. The LSFG measurements correlate strongly with those obtained using hydrogen gas clearance and microsphere methods.^11^^,^^12^ Previous research using LSFG to measure retinal blood flow in DR has shown reduced vascular blood flow in proliferative DR.^13^ Furthermore, ranibizumab intravitreal injection has been reported to significantly reduce retinal blood flow in patients with DME.^14^^,^^15^ LSFG provides a parameter known as total capillary resistance (TCR), which reflects vascular resistance.^16^ Elevated retinal arterial vascular resistance has been reported in the branch retinal vein occlusion area^17^ and in central retinal vein occlusion (CRVO).^16^ However, vascular resistance in diabetic retinopathy remains underevaluated.

In this study, we measured retinal blood flow and vascular resistance in both normal eyes and eyes with DR and compared the results.

## Methods

This retrospective observational single-center case-control study was conducted in accordance with the Declaration of Helsinki and approved by the Ethics Committee of Kagawa University, Kagawa, Japan.

A total of 224 patients with DR who visited the Department of Ophthalmology, Kagawa University Hospital, between April 2019 and May 2023 were initially considered. After applying the exclusion criteria, 87 eyes from 87 patients (60 males, 27 females; mean age: 62.2 ± 11.3 years) were included. In total, 87 eyes with DR and 50 control eyes from 50 patients without DR (36 males, 14 females; mean age: 60.3 ± 9.2 years) were analyzed. All patients were Japanese.

The patients underwent blood pressure measurements using an automated sphygmomanometer (TM-2657p; A&D, Tokyo, Japan), best-corrected visual acuity, intraocular pressure, and ocular perfusion pressure (calculated as two-thirds average artery pressure minus intraocular pressure), as well as slit-lamp microscopy (SL130; Carl Zeiss, Jena, Germany) and fundus examination under mydriasis. Optical coherence tomography (SPECTRALIS HRA+OCT; Heidelberg Engineering, Heidelberg, Germany) was used to measure central foveal retinal thickness, while LSFG was used to measure retinal blood flow and retinal vascular resistance. Patients diagnosed with DR were classified according to the International Classification of Diabetic Retinopathy Severity.^18^ These patients were compared to age-matched controls without diabetes.

### Exclusion Criteria

Patients with the following cases were excluded: age-related macular degeneration, central serous chorioretinopathy, glaucoma, uveitis, vitrectomy at any time previously, subtenon triamcinolone acetonide injection or vitreous injection of anti–vascular endothelial growth factor (VEGF) drugs within the past 1 year, vitreous hemorrhage, proliferative membrane over the optic nerve papilla, photocoagulation within the past 1 year, severe cataracts, and cases of missing data. The breakdown of excluded cases is shown in Table 1.

**Table 1.** Details of Excluded Cases (*n* = 137)

Exclusion Criteria	*n* (%)
Vitreous hemorrhage	30 (21.9)
Cases of missing data	25 (18.2)
Glaucoma	17 (12.4)
Vitrectomy at any time previously	13 (9.5)
Severe cataracts	12 (8.8)
Vitreous injection of anti-VEGF drugs within 1 year	11 (8.0)
Proliferative membrane over the optic nerve papilla	8 (5.8)
Photocoagulation within 1 year	8 (5.8)
STTA within 1 year	5 (3.6)
Age-related macular degeneration	4 (2.9)
Central serous chorioretinopathy	3 (2.2)
Uveitis	1 (0.7)

STTA, subcapsular injection of triamcinolone acetonide; VEGF, vascular endothelial growth factor.

### Measurement by LSFG

**Figure 2.**
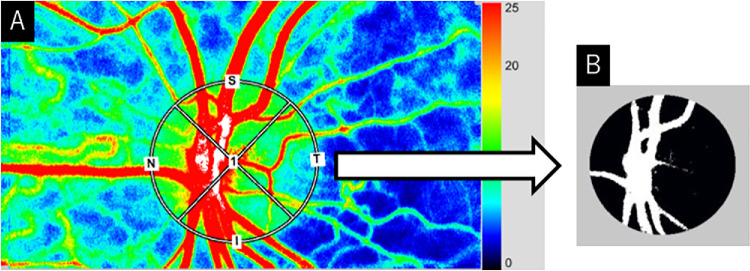
The sample image shows how MV and MT were measured. (**A**) The color map of the total measurement area, with the vascular and tissue areas in the optic papillary region distinguished by the vascular extraction function of the software (**B**).

**Figure 3.**
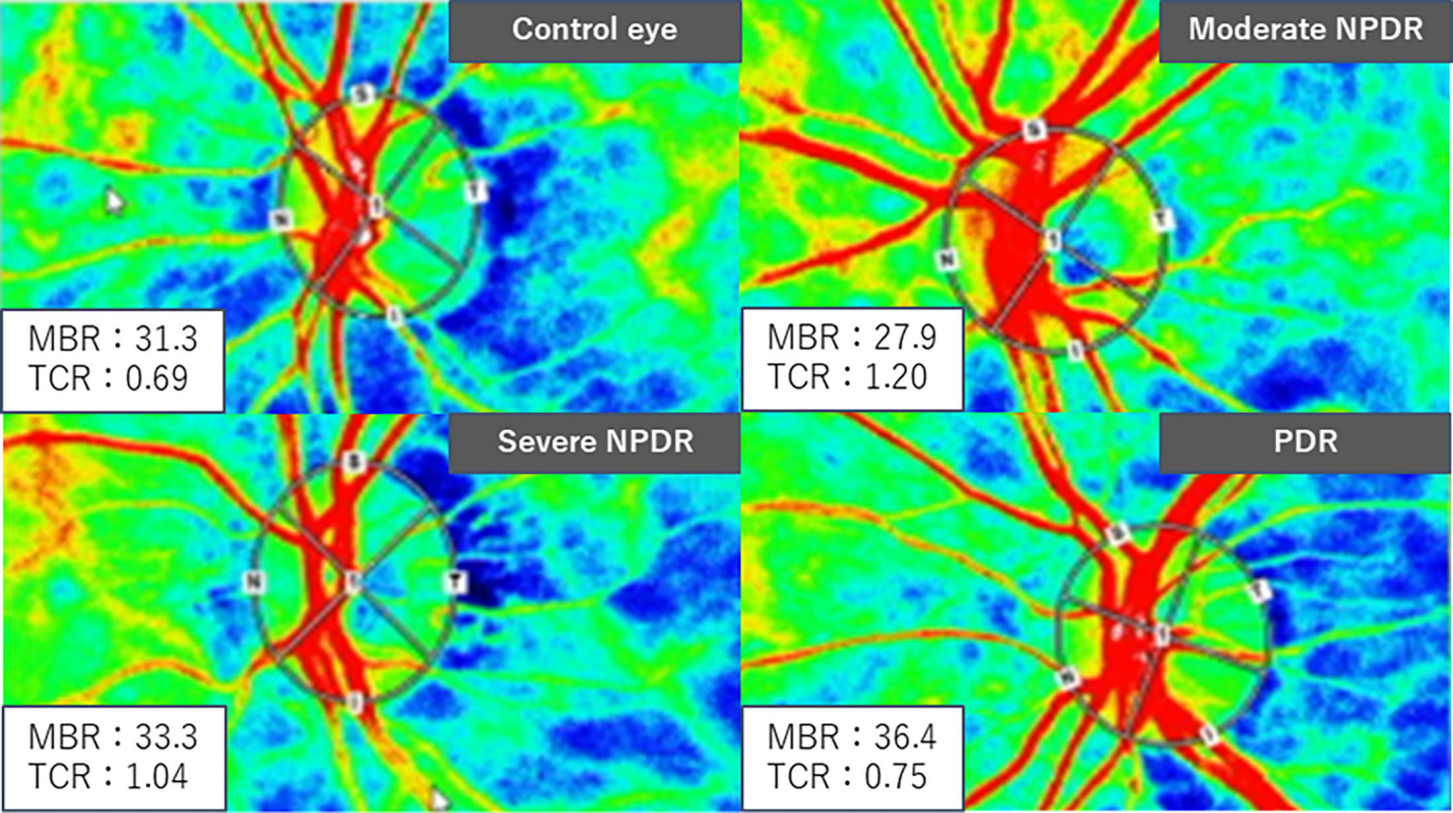
Typical LSFV-NAVI images of each group.

LSFG-NAVI (Softcare, Fukuoka, Japan) was used to measure the mean blur rate (MBR) in the optic papillary region. From the color map of total measurement area (Fig. 2A), the vascular and tissue areas within the optic papillary region were distinguished using the software's vascular extraction function (Fig. 2B). The MBR in both compartments could then be evaluated independently. Since the MBR of the optic papillary vascular area (MV) includes choroidal blood flow, the MV minus the MBR of the tissue area (MT) was used to measure blood flow in the optic papillary great vessels. In other words, we calculated the value by subtracting the black area (MT) from the white area (MV) in Figure 2B. The principle of LSFG has been described in detail in previous reports.^9^^,^^10^^,^^19^ Representative analysis images for each LSFG-NAVI group are shown in Figure 3.

MBR was measured as pulsatile blood flow over a 4-second cardiac cycle, and beat strength (BS) was calculated based on the amplitude between the maximum and minimum blood flow. BS is defined as^20^

$$\begin{array}{c} BS=C\cdot \max_{i}P\left(fi\right) \end{array}$$

where *C* is a constant scaling factor and *P*(*fi*) is the power spectrum calculated from the time-series data of the optic papillary great vessels, in the same region as MV-MT is calculated. The detailed formula for calculating BS has been published previously.^20^^,^^21^ TCR, a parameter that reflects the resistivity of the entire retinal vasculature, is defined by the following equation:^16^

$$\begin{array}{c} \mathrm{TCR}\;=\mathrm{BS}/\left(\mathrm{MV}-\mathrm{MT}\right) \end{array}$$

TCR represents the total resistivity of all retinal vessels, including retinal arterioles, microvessels, capillaries, and central retinal veins.

### Statistical Analysis

The values for each parameter are expressed as the mean ± standard deviation. Comparisons between groups were performed using the χ^2^ test and analysis of variance. Additionally, a receiver operating characteristic (ROC) curve was used to assess the diagnostic performance of the TCR in the presence or absence of DR. The area under the ROC curve was used to evaluate the diagnostic potential of TCR to identify DR, and the Youden index determined the cutoff value. All statistical analyses were performed using R (R Foundation for Statistical Computing, Vienna, Austria), with a significance level set at *P* < 0.05.

## Results

The clinical characteristics of each group are presented in Table 2. Sex distribution did not differ significantly among the groups. Age was significantly higher in the moderate NPDR group and lower in the PDR group compared to the control eyes. The mean hemoglobin A1c level in the DR group was 7.9% ± 1.9%, with no significant differences across the International Clinical Severity Scales. Moreover, the mean blood pressure was significantly lower in the moderate NPDR group compared to control eyes. Post–cataract surgery was more prevalent in the severe NPDR group than in the control group. No significant differences were observed in hypertension, ischemic heart disease, or cerebral infarction incidence. Severe NPDR was the most common condition, affecting 44 (50.6%) eyes. In total, 31 eyes underwent photocoagulation, including 11 with focal photocoagulation and 20 with panretinal photocoagulation (PRP). All patients who underwent photocoagulation had severe NPDR or higher.

**Table 2.** Clinical Characteristics of Each Group

Characteristic	Control Eyes (*n* = 50)	DR Eyes (*n* = 87)	Moderate NPDR (*n* = 17)	Severe NPDR (*n* = 44)	PDR (*n* = 26)
Men/women, *n*	36/14	60/27	10/7	28/16	22/4
Age, y	60.3 ± 9.2	62.2 ± 11.3	70.8 ± 7.8^*^	64.6 ± 8.8	52.3 ± 9.9^*^
Hemglobin A1c, %	—	7.9 ± 1.9	7.2 ± 0.9	7.8 ± 1.7	8.7 ± 2.5
Average duration of diabetes, mo	—	170.1 ± 126.0	218.7 ± 129.9	168.8 ± 130.6	150.0 ± 110.6
SBP, mm Hg	137.8 ± 20.0	137.7 ± 18.7	132.5 ± 17.7	138.8 ± 16.3	138.7 ± 22.2
DBP, mm Hg	84.4 ± 12.7	75.6 ± 12.0	70.6 ± 10.8	74.5 ± 10.4	80.4 ± 13.5
Mean blood pressure, mm Hg	102.2 ± 13.7	96.3 ± 12.5	91.2 ± 11.4^*^	95.9 ± 10.5	99.8 ± 14.8
History					
IOL	4 (8)	24 (27.6)	5 (29.4)	17 (38.6)^*^	2 (7.7)
Hypertension	18 (36.0)	52 (59.8)	7 (41.2)	28 (63.6)	16 (61.5)
Hyperlipidemia	8 (16.0)	24 (27.6)	6 (35.3)	6 (13.6)	11 (42.3)
Ischemic heart disease	1 (2.0)	5 (5.7)	0 (0)	2 (4.5)	3 (11.5)
Cerebral infarction	1 (2.0)	5 (5.7)	1 (5.9)	0 (0)	4 (15.4)
Photocoagulation					
No photocoagulation	—	56 (64.4)	17	26	13
Focal PRP	—	11 (12.6)	0	10	1
PRP	—	20 (23.0)	0	8	12
Diabetic macular edema	52 (59.8)	10 (58.8)	32 (72.7)	10 (38.5)	

DBP, diastolic blood pressure; NPDR, non-proliferative diabetic retinopathy; PDR, proliferative diabetic retinopathy; SBP, systolic blood pressure.

Values are expressed as the mean ± SD or *n* (%) unless otherwise indicated.

* Significant (*P* < 0.05) vs. control eyes.

**Figure 4.**
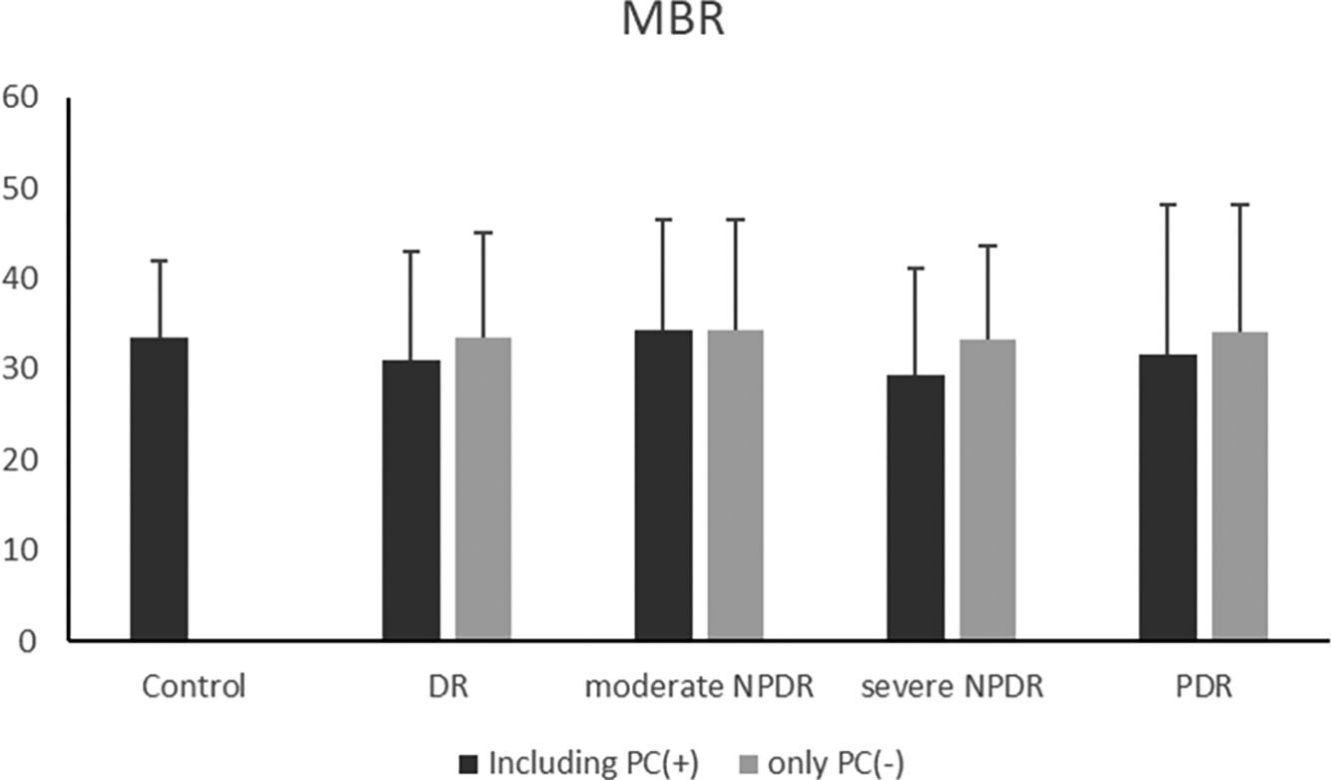
MBR of control and DR eyes at each stage of retinopathy. We compared each group, excluding retinal photocoagulation cases, and found no significant difference between normal eyes and the groups.

**Figure 5.**
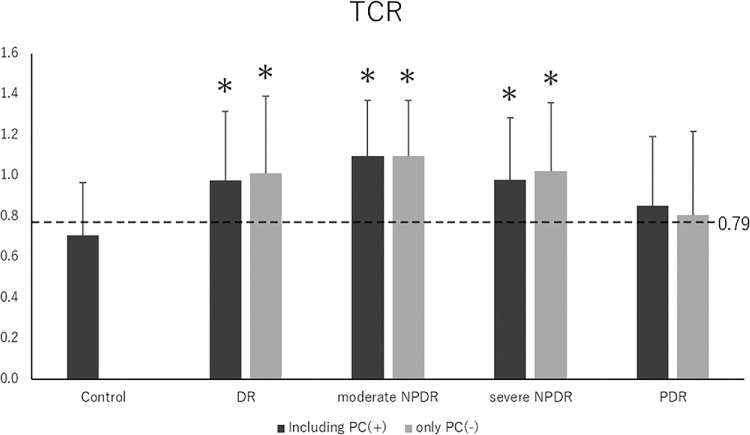
TCR of control and DR eyes at each stage of retinopathy. We compared each group, excluding retinal photocoagulation cases. TCR was higher in DR, moderate in NPDR, and severe in NPDR eyes compared to control eyes (*P* < 0.01, analysis of variance, *P < 0.01). A similar trend was observed with and without retinal photocoagulation. The cutoff value for TCR was 0.79.

The measured values for each group are presented in Table 3. Best-corrected visual acuity was reduced in all groups except the PDR group. Central retinal thickness was elevated in the entire DR group (449.8 ± 84.8) and the severe NPDR group (396.3 ± 111.0). However, MBR did not differ significantly between normal eyes and the other groups. Notably, TCR was significantly higher in all groups, except the PDR, compared to normal eyes. MBR and TCR were compared between groups, excluding cases that underwent retinal photocoagulation (Figs. 4, 5). The MBR and TCR values, excluding cases with retinal photocoagulation, are shown in the order of DR, severe NPDR, and PDR. MBR values were 33.4 ± 9.4, 33.3 ± 8.0, and 34.04 ± 11.9, respectively, while TCR values were 1.01 ± 0.38, 1.02 ± 0.34, and 0.81 ± 0.41, respectively. The trends were similar with and without retinal photocoagulation.

**Table 3.** Measured Values of Each Group

Characteristic	Control Eyes (*n* = 50)	DR Eyes (*n* = 87)	Moderate NPDR (*n* = 17)	Severe NPDR (*n* = 44)	PDR (*n* = 26)
BCVA, logMAR	−0.091 ± 0.10	0.31 ± 0.21^**^	0.17 ± 0.21^**^	0.23 ± 0.23^**^	0.13 ± 0.29
IOP, mm Hg	16.4 ± 2.7	15.6 ± 2.9	14.2 ± 2.2	15.6 ± 2.8	16.3 ± 3.0
OPP, mm Hg	51.7 ± 5.6	48.8 ± 6.8	46.9 ± 6.9	48.4 ± 6.9	50.2 ± 8.9
CRT, µm	283.0 ± 27.8	380.8 ± 112.9^**^	369.1 ± 95.5	396.3 ± 111.0^**^	362.3 ± 122.5
MBR	33.4 ± 6.9	31.0 ± 10.5	34.4 ± 9.08	29.4 ± 8.9	31.6 ± 9.9
TCR	0.71 ± 0.26	0.95 ± 0.3^**^	1.10 ± 0.27^**^	0.98 ± 0.3^**^	0.85 ± 0.34

BCVA, best-corrected visual acuity; CRT, central retinal thickness; IOP, intraocular pressure; MBR, mean blur rate; OPP, ocular perfusion pressure = 2/3 (average artery pressure) – IOP; TCR, total capillary resistance.

Values are expressed as the mean ± SD.

** Significant (*P* < 0.01) vs. control eyes.

**Figure 6.**
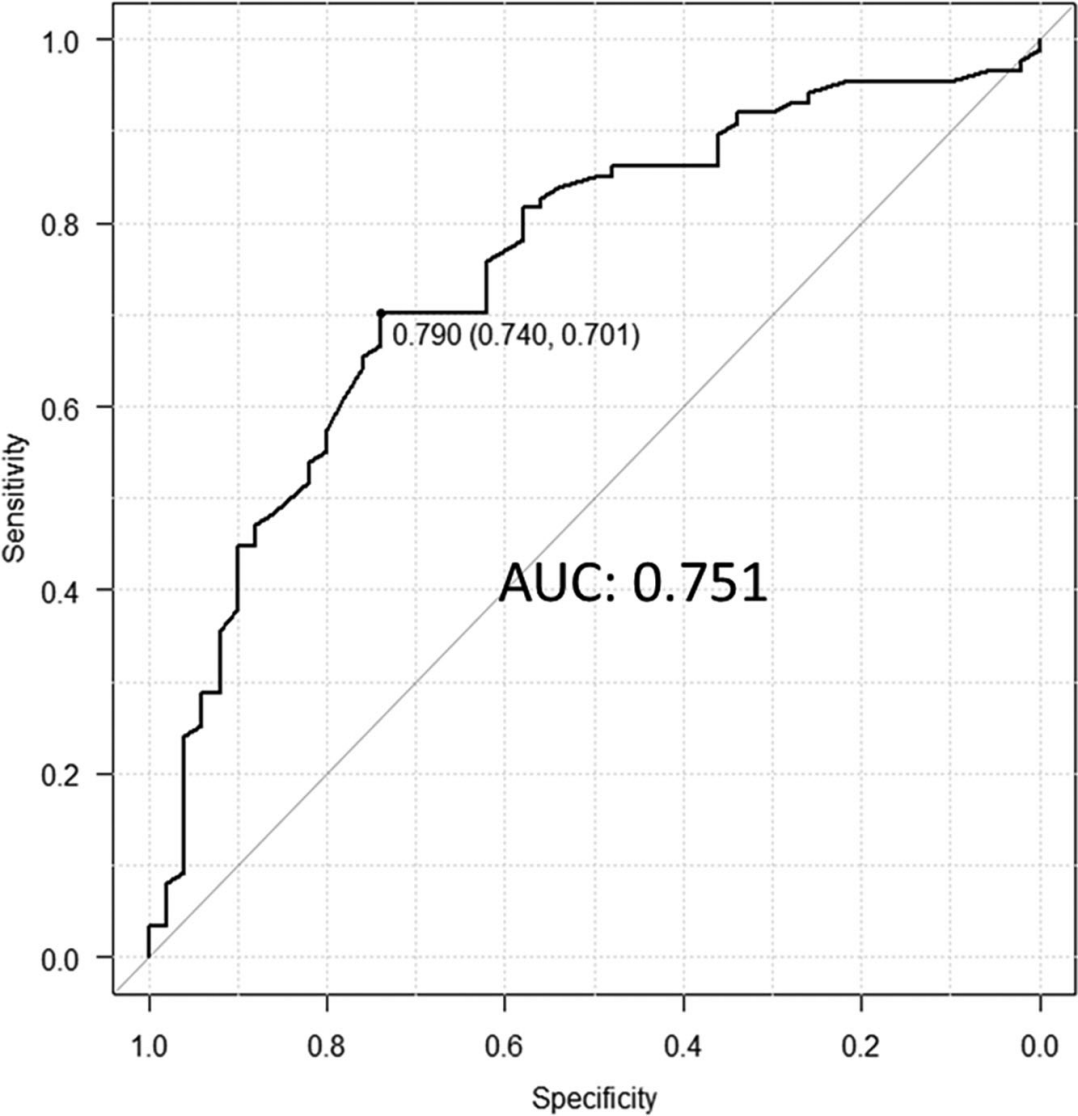
ROC curves of TCR in control and DR eyes. Using the Youden index, the TCR cutoff value was 0.79 (sensitivity, 0.740; specificity, 0.701). The area under the ROC curve was 0.751.

The ROC curve was used to assess the diagnostic performance of TCR for DR (Fig. 6). The TCR area under the ROC curve was 0.751, indicating moderate diagnostic accuracy for DR. Using the Youden index, the TCR cutoff value was 0.79 (sensitivity, 0.740; specificity, 0.701). The TCR values for each group are shown in Figure 5. The cutoff value for TCR was between the mean values of the control eyes and the PDR groups.

## Discussion

In this study, patients in the moderate NPDR group were older, while those in the PDR group were younger, compared to the control eye group. The severity of retinopathy has been associated with a younger age at diabetes diagnosis,^22^ consistent with previous reports. Additionally, the prevalence of post–cataract surgery was higher in the severe NPDR group than in the control group. Since patients with diabetes are known to develop cataracts more frequently and at an earlier age than patients without diabetes,^23^ this may explain the higher prevalence of intraocular lens implantation in patients with DR.

MBR did not differ significantly between normal and DR eyes, nor were significant differences observed when comparing DR across stages. This finding contrasts with a previous study that reported reduced blood flow in patients with DR using the laser Doppler technique in the NDR and mild NPDR groups compared to the nondiabetic group.^7^ One major explanation for this discrepancy is that the present study included a large number of patients with DME and those who had undergone photocoagulation. DME eyes may be affected by increased vascular permeability and vasodilation due to VEGF activity, while PRP has been reported to decrease retinal blood flow.^24^^–^^26^ The effect of blood pressure on retinal blood flow is also of concern, as DR is associated with impaired autoregulation of retinal blood flow.^27^^,^^28^ Given that multiple factors can affect retinal blood flow, no significant difference may have been observed in this study.

Notably, TCR was significantly higher in eyes with DR than in normal eyes. The only previous report on TCR in humans examined CRVO.^16^^,^^29^ To our knowledge, no studies have investigated TCR in the context of DR. In DR, pericytes are lost due to hyperglycemia and oxidative stress, leading to vascular endothelial cells damage, and increased leukocyte adhesion to vessel walls, with elevated expression of adhesion molecules, ultimately resulting in capillary occlusion.^30^ In a rat model of streptozotocin-induced diabetes, diabetic retinal vascular leakage and nonperfusion were temporally and spatially associated with retinal leukocyte stasis (leukostasis).^31^ Retinal leukostasis correlates with increased expression of retinal intercellular adhesion molecule 1.^31^ Blocking intercellular adhesion molecule 1 with a monoclonal antibody prevents diabetic retinal leukostasis and vascular leakage by 48.5% and 85.6%, respectively.^31^ We hypothesized that the elevated TCR in the eyes with DR may be due to leukostasis.

DR is a major complication of diabetes, making the prediction of its development and progression clinically crucial. The cutoff value for TCR in CRVO was reported to be 0.93,^16^ higher than that observed in DR in this study. The area under the curve was 0.751, indicating that TCR may serve as a moderate predictive potential biomarker for DR. Assessing retinal vascular resistance may provide insight into the early pathophysiology of DR and help predict its development. Further study is necessary to investigate the change in each DR stage.

A limitation of this study is that it cannot determine whether the increase in TCR of vascular resistance is due to diabetes or DR, as patients with diabetes and no DR or mild NPDR were excluded, while those with DME and those who underwent photocoagulation were included. Additionally, all participants were Japanese, and the design was based on a single institution, limiting the study's generalizability. Therefore, further studies are needed to clarify the exact pathogenesis of elevated TCR.
